# Exploring the relationship between gratitude and depression among older adults with chronic low back pain: a sequential mediation analysis

**DOI:** 10.3389/fpain.2023.1140778

**Published:** 2023-05-05

**Authors:** Melissa Makhoul, E. J. Bartley

**Affiliations:** ^1^ Hariri School of Nursing, American University of Beirut, Beirut, Lebanon; ^2^ Department of Community Dentistry and Behavioral Science, University of Florida, Gainesville, FL, United States

**Keywords:** gratitude, depression, well-being, older adults, chronic low back pain

## Abstract

**Introduction:**

Gratitude has been identified as a key factor in a number of positive health-related outcomes; however, the mechanisms whereby gratitude is associated with well-being among older adults with chronic pain are poorly understood. Using the Positive Psychological Well-Being Model as a theoretical framework, the objective of the present study was to examine the serial mediating effects of social support, stress, sleep, and tumor necrosis factor-alpha (TNF-α) on the relationship between gratitude and depressive symptoms.

**Methods:**

A total sample of 60 community-dwelling older adults with chronic low back pain (cLBP) provided blood samples for high-sensitivity TNF-α and completed the Gratitude Questionnaire, Perceived Stress Scale, and the PROMIS Emotional Support, Sleep Disturbance, and Depression forms. Descriptive statistics, correlation analyses, and serial mediation analyses were performed.

**Results:**

Gratitude was negatively associated with perceived stress, sleep disturbance, and depression, and was positively associated with social support. No significant association was observed between gratitude and TNF-α. After controlling for age and marital status, analyses revealed that perceived stress and sleep disturbance sequentially mediated the association between gratitude and depressive symptoms.

**Conclusion:**

Perceived stress and sleep disturbance may be potential mechanistic pathways by which gratitude impacts negative well-being. Targeting gratitude as a protective resource may be a potential therapeutic tool to improve psychological and behavioral outcomes in older adults with cLBP.

## Introduction

Low back pain is one of the most common and disabling conditions among older adults (1). Studies estimate that the 12-month prevalence of chronic low back pain (cLBP) ranges from 21% to 75% in individuals aged 60 years or older (2), and 36.1% in community-dwelling older adults (3). While the pathogenesis of cLBP is unknown, a number of biopsychosocial factors are implicated in its etiology and play key roles in the development and maintenance of disabling low back pain (4, 5). One of the most prevalent psychosocial factors in older age is depression, with a prevalence rate of 31.7% (6) and an estimated 15.7% of older adults with chronic pain have comorbid depressed mood (7). Although depression is commonly considered a consequence of chronic pain, their relationship is bidirectional (8), with both exacerbating one another resulting in poorer physical, mental, and social health relative to individuals experiencing pain or depression alone (9). Sleep disturbances are also highly prevalent among older adults with chronic pain, with more than 50% reporting at least one problem related to sleep duration or quality (10). Evidence also suggests that older adults with chronic pain commonly experience stress, which in turn has been associated with greater levels of pain intensity and interference (11). In addition, heightened levels of systemic inflammatory cytokines, including tumor necrosis factor alpha (TNF-α), have been observed in individuals with cLBP, relative to patients with acute back pain and healthy controls (12), and evidence suggests a significant association between TNF-α and increasing levels of pain severity among those with chronic pain (12, 13). Despite being a highly prevalent and disabling condition among older adults, cLBP is often undertreated among this cohort (14).

Emerging evidence suggests that older adults have the capacity for resilience and experience positive outcomes such as high well-being, despite the presence of chronic conditions such as pain (15, 16). One positive psychological construct that may promote resilience in older adults is gratitude (17), which broadly refers to a state of appreciation and/or thankfulness of things one has in life (18). A large body of evidence suggests that gratitude is beneficial to aspects of well-being (18–20); however, little is known regarding the potential mechanisms underlying this association. While earlier frameworks provide possible mechanisms by which gratitude may be linked with well-being (21), an adaptation of the positive psychological well-being (PPWB) model (22) has been recently proposed (23). Unlike previous theoretical models which assume that the absence of unhealthy behaviors or disease-related biological markers is indicative of health, the PPWB model posits that positive psychological well-being, such as gratitude, has its own independent association with promoting health. This relationship is mediated by the presence of restorative processes and the absence or reduction of deteriorative processes which includes many behavioral and biological factors (23). In line with this model, gratitude may directly influence engagement in restorative health behaviors (e.g., obtaining optimal sleep) and impact markers of restorative biological functioning (e.g., serum antioxidants). In addition, gratitude is expected to be associated with lower engagement in deteriorative health behaviors (e.g., tobacco use), as well as lower biological dysfunction (e.g., inflammation) (22). Supporting this theoretical framework, gratitude has been linked to reduced inflammatory cytokines, such as TNF-α, that are known to predict adverse cardiovascular outcomes, as well as adherence to healthy behaviors such as exercise, diet, and better sleeping patterns (24). In the context of chronic pain, gratitude has been found predictive of lower depression through better sleep quality (25). This is particularly important in individuals with chronic pain as poor sleep exacerbates the pain experience (26).

Other components of the PPWB model relate to the role of stress and social support, such that gratitude could indirectly improve health behaviors and biological functioning through a reduction in stress and increase in levels of social support. Aligning with this, there is evidence suggesting an indirect effect of gratitude on inflammatory markers (TNF-α) via increases in support giving (27), and studies have shown that grateful individuals are more likely to perceive and receive greater social support (28, 29), which in turn enhances the positive effects of social support on psychological well-being (30). This is especially salient to older adults as this population is more vulnerable to experiencing loneliness and social isolation, largely due to personal losses, poor health conditions, and living alone (31, 32), and cLBP may further restrict their ability to participate in social activities (33). Subsequently, social isolation and loneliness increase the risk of depression and mortality (31, 34), and evidence suggests that gratitude diminishes levels of perceived stress over time (35). As perceived stress has been shown to be independently associated with poor sleep quality among older adults with chronic pain (10), gratitude may lead to improvements in health through reductions in stress (19). For instance, one study demonstrated an indirect effect of gratitude on subjective well-being via decreases in levels of perceived stress (36).

**Figure 1:**
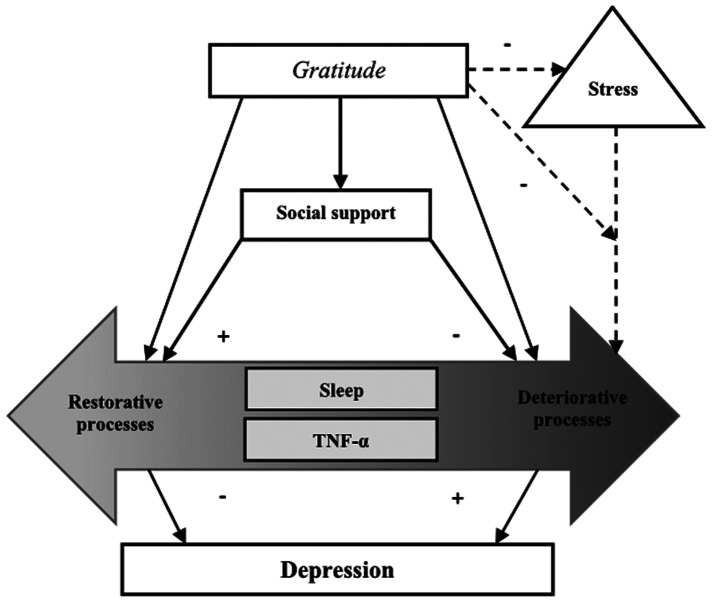
Theoretical model of positive psychological well-being (PPWB) demonstrating the pathways through which gratitude is related to depression. This model proposes that increased gratitude directly influences sleep disturbance and TNF-α as well as indirectly through social support and stress. [PPWB Model by Boehm and Kubzansky (2012) adapted with permission].

Although findings have shown that gratitude is associated with better psychological well-being, few studies have evaluated the mechanisms explaining how gratitude influences health outcomes. Because older adults are at increased risk for depression, examining gratitude and its association with depression along with its potential mediators may guide the development and implementation of gratitude and other positive psychological interventions to improve health outcomes in aging adults with cLBP. Based on the adapted PPWB model (22, 23), the purpose of this study was to empirically test a theoretical model in which we examine whether self-reported social support, stress, sleep disturbance, and TNF-α serially mediate the relationship between gratitude and depressive symptoms in a sample of older adults with cLBP (Figure 1). We hypothesized that: (1) higher levels of gratitude would be associated with greater social support and lower levels of perceived stress, sleep disturbance, TNF-α, and depression, and (2) social support, stress, sleep disturbance, and TNF-α would serially mediate the association between gratitude and depressive symptoms.

## Materials and methods

### Participants

This was a cross-sectional study involving a secondary data analysis using the same population from the Adaptability and Resilience in Aging Adults (ARIAA) study, originally designed to examine the effects of resilience mechanisms on pain among individuals with chronic low back pain (cLBP). A detailed description of the screening and inclusion/exclusion criteria has been previously published (15, 16). For the original study, ethics approval was provided by the University of Florida (UF) Institutional Review Board (IRB) and all participants provided verbal and written informed consent prior to study procedures. The sample consisted of 69 older adults (aged 60 years and above) with cLBP. Participants were recruited from the community via posted fliers, radio and print media announcements, and word-of-mouth referral. Participants were older adults aged 60 years and above (37), and were included if they reported a minimum of mild low back pain (LBP) of ≥2/10 on the numeric rating scale (NRS) for at least half of the days during the past three months. Key exclusion criteria included: (1) recent vertebral fracture, (2) undergoing back surgery within the past six months, (3) diagnosis of cauda equina syndrome, (4) uncontrolled hypertension (≥150/90), (5) current severe cardiovascular disease, (6) neurological diseases associated with somatosensory abnormalities (e.g., neuropathy, seizures, Parkinson's disease), (7) current major medical illness (e.g., metastatic or visceral disease), (8) chronic opioid use, and (9) systemic inflammatory disease (e.g., spondyloarthropathies).

### Procedures

Participants were initially evaluated for study eligibility through a brief telephone screen. As part of the screening process, participants completed baseline questionnaires covering sociodemographic information that included age, sex, race, marital and employment status, education level, annual income, back pain duration; as well as a brief health history involving the presence of major medical illnesses, recent back-related injuries or surgeries, and LBP symptoms. If eligible, participants attended two study visits lasting 2–3.5 h each and held approximately one week apart. During the first visit, study eligibility was verified via self-reported demographic and medical history assessment, and participants provided informed consent in compliance with the Declaration of Helsinki. In addition, participants completed anthropometric tests, psychosocial questionnaires, and functional performance measures. Participants also completed a battery of self-report questionnaires at home between visit 1 and 2. During the second visit, blood samples were collected prior to the initiation of any study procedures, followed by the completion of additional psychosocial questionnaires. Participants were provided up to a $100 honorarium after study completion.

### Measures

#### Gratitude (Gq-6)

Gratitude was examined using the Gratitude Questionnaire GQ-6 (38), which is a brief six-item self-report measure to assess one's disposition towards experiencing gratitude in daily life (e.g., “I am grateful to a wide variety of people”). Responses are provided using a seven-point scale (1 = strongly disagree, 7 = strongly agree). The total scores range from 6 to 42, with higher scores indicating greater gratitude. The GQ-6 produces a single-factor score and has convergent validity with other gratitude measures (38). The GQ-6 has been translated to many languages, each of which demonstrated good psychometric properties (39–41). In this sample, the Cronbach's *α* for GQ-6 was 0.86.

#### PROMIS emotional support

To assess social functioning, the PROMIS Social Relationships Short Forms (PROMIS-SR) were administered (43, 44), which contain eight items on emotional support (e.g., “I have someone who will listen to me when I need to talk”). Items are scored using a 5-point Likert-type scale (1 = never, 5 = always). The total scores range from 8 to 40, with higher scores indicating greater social support. The English version of the PROMIS-SR has shown adequate reliability and validity, and its psychometric properties were comparable with versions in other languages (43, 45). In the present study, Cronbach's alpha of the PROMIS-Emotional Support was 0.97.

#### Perceived stress scale

Perceived stress was assessed using the Perceived Stress Scale (PSS), which is a brief 10-item self-report measure to assess the degree to which situations in one's life are perceived as stressful (e.g., “In the last month, how often have you felt nervous and stressed”) (46). Responses are provided using a four-point scale (0 = never, 4 = very often). A total perceived stress score ranging from 0 to 40 is computed, with higher scores indicating higher perceived stress. Various studies suggest that the psychometric properties of the PSS-10 are satisfactory (47). In this sample, the Cronbach's *α* for the PSS was 0.92.

#### PROMIS sleep disturbance

Sleep disturbance was measured using the PROMIS Sleep Disturbance Short Form (48), which is an eight-item self-report measure to assess difficulties falling and staying asleep (e.g., “My sleep was restless”). Respondents rate aspects of their sleep in the past seven days on a 5-point Likert scale (1 = not at all, 5 = very much). The total scores range from 8 to 40, with higher scores indicating greater sleep disturbance. In the present study, the PROMIS Sleep Disturbance Short Form demonstrated adequate reliability (Cronbach's *α* = 0.93), which is consistent with published norms (Cronbach's *α* > 0.90) (48, 49).

#### Inflammatory measures (TNF-α)

Venous blood samples were collected in EDTA-Vacutainer® tubes (#V T-6450) by a study nurse. Immediately following collection of the sample, the blood was centrifuged at 1,600×*g* for 15 min at 4 °C, aliquoted, and stored at −80 °C until analysis. All assays were analyzed in duplicate with a high-sensitivity, commercially available multiplex immunoassay panel (e.g., MILLIPLEX Multi-Analyte Panels). All samples were collected within a two-hour window (between 8:00 am to 10:00 am), and participants were asked to refrain from food, beverage, and nicotine consumption for 3 h; exercise for 4 h; and alcohol for 12 h prior to their study visit. Results are reported in pg/ml.

#### PROMIS depression scale

Depression was measured using the eight-item PROMIS-Depression Short Form (e.g., “I feel worthless”) (50). Respondents rate the frequency of their experience of each depressive symptom in the past seven days on a 5-point Likert scale (1 = never, 5 = always). Total scores range from 8 to 40, with higher scores indicating a greater presence of depressive symptoms. The PROMIS Depression Short form has been shown to have high reliability (Cronbach's *α* = 0.98) (49), which was excellent within this sample (Cronbach's *α* = 0.93).

### Data analysis

All statistical analyses were carried out using the Statistical Product and Service Solution (SPSS) version 25.0. Preliminary analysis examined the presence of outliers and missing values, and the assumptions of normality were met. The existing literature indicates that depression has numerous correlates and varies across sociodemographic backgrounds (e.g., age, sex, education, employment, socioeconomic status, marital status) (51, 52). To identify potential covariates, zero-order correlation analyses were conducted between sociodemographic characteristics and the outcome variable (i.e., depression). Sociodemographic variables that were significantly related to depression were controlled for in all mediation analyses.

Hayes' PROCESS macro (Model 6) (53) was used to examine the serial-multiple mediator model. Four hypothesized multiple serial-mediation models were explored in order to analyze the indirect effects of gratitude on depression (Model 1 mediators: social support-sleep disturbance; Model 2 mediators: social support-TNF-α; Model 3 mediators: perceived stress-sleep disturbance; Model 4 mediators: perceived stress-TNF-α). A serial mediation model assumes one mediator affects another mediator within a specified direction, such that gratitude (X) could increase levels of social support (mediator 1), which could decrease sleep disturbance (mediator 2), resulting in lower levels of depression (Y). The total effect of X on Y is denoted as *c*, which consists of the sum of one direct effect represented by path *c*′ and three indirect effects (*a*_1_*b*_2_, *a*_2_*d*_1_, *a*_1_*b*_1_*d*_1_). The 95% bias-corrected bootstrap confidence interval was based on 10,000 bootstrap samples to generate the path estimates and the indirect effects with a 0.05 criterion for rejection (two-tailed). Results were statistically significant when zero was not contained in the bootstrap 95% CIs.

## Results

### Participants' characteristics

Sociodemographic characteristics of participants and descriptive statistics of all study measures are presented in Table 1. Participants were mostly white (70%), females (57%), had a college degree (50%), were married or partnered (52%), unemployed (85%), and had an income of ≤$20,000 (36.8%). Average age was 68 years (range: 60–93 years), and duration of back pain was 16.4 years (range: 1–56 years). Two of the 69 participants discontinued after the first session due to time constraints, and seven participants who were initially eligible were excluded during their first appointment (*n* = 1 use of exclusion medications, *n* = 3 exclusionary medical condition, *n* = 3 not meeting pain duration criteria), thereby leaving 60 participants.

**Table 1:** Sample demographics and descriptive statistics of study measures.

Variable	*N* (%)	Mean (SD)
Age (years)		68.1 (7.0)
**Sex**		
Male	26 (43.3)	
**Race**		
White	42 (70.0)	
Black	12 (20.0)	
Other	6 (10.0)	
**Education**		
≤High school diploma	13 (21.7)	
Some college/technical degree	17 (28.3)	
College degree	18 (30.0)	
Graduate/professional	12 (20.0)	
**Marital status**		
Not married	29 (48.3)	
**Employment**		
Employed	9 (15.0)	
**Income**		
<$20,000	21 (36.8)	
$20,000–39,999	10 (17.5)	
$40,000–59,999	11 (19.3)	
$60,000–99,999	8 (14.0)	
≥$100,000	7 (12.3)	
Back pain duration (years)		16.4 (14.2)
Body mass index (BMI)		29.3 (5.8)
**Study Measures**		
GQ-6		35.4 (6.8)
PROMIS-Emotional support		31.0 (8.3)
PROMIS-Sleep disturbance		20.6 (8.2)
PROMIS-Depression		13.1 (5.9)
PSS		13.6 (7.9)
TNF-α (pg/ml)		6.5 (2.2)

GQ, Gratitude Questionnaire; PROMIS, Patient-Reported Outcomes Measurement Information System; PSS, Perceived Stress Scale; TNF, Tumor Necrosis Factor.

### Zero-Order correlations

Table 2 presents zero-order correlations of sociodemographic characteristics with gratitude, emotional support, sleep disturbance, perceived stress, TNF-α, and depressive symptoms. Gratitude was positively associated with emotional support (*r* = 0.39, *p* = 0.002), and was negatively associated with sleep disturbance (*r* = − 0.38, *p* = 0.003), perceived stress (*r* = − 0.40, *p* = 0.002), and depression (*r* = − 0.41, *p *= 0.001). There was no association observed between gratitude and TNF-α (*r* = 0.13, *p* = 0.344). Age (*r* = − 0.31, *p* = 0.017) and marital status (*r* = 0.31, *p* = 0.018) were moderately associated with depression and were included as covariates in all mediation analyses to control for their potential effects.

**Table 2:** Zero-Order correlations among study variables.

Variable	1	2	3	4	5	6
Age	0.07	0.08	0.25	−0.43**	0.33*	−0.31*
Sex	−0.18	0.17	0.06	−0.12	0.23	−0.10
Race	−0.10	0.11	0.18	0.08	0.03	0.02
Education	0.10	0.15	−0.19	−0.02	−0.12	0.11
Marital status	−0.17	−0.47**	0.36**	0.37**	0.07	0.31*
Income	0.27*	0.28*	−0.23	−0.22	0.11	−0.22
Back pain duration	0.03	0.13	−0.06	−0.14	0.01	−0.15
Body Mass Index	−0.13	−0.18	0.24	0.08	−0.06	0.21
1. GQ-6	1	0.39**	−0.38**	−0.40**	0.13	−0.41**
2. PROMIS-Emotional support		1	−0.43**	−0.49**	0.06	−0.66**
3. PROMIS-Sleep disturbance			1	0.55**	−0.14	0.62**
4. PSS				1	−0.08	0.78**
5. TNF-α					1	−0.20
6. PROMIS-Depression						1

Sex coded: 0 = female, 1 = male; Race coded: 0 = NHW, 1 = NHB; Education coded: 0 = ≤high school degree, 1 = > high school degree; Marital status coded: 0 = married, 1 = not married; Income coded: 0 = <$20,000, 1 = ≥$20,000. GQ, Gratitude Questionnaire; PROMIS, Patient-Reported Outcomes Measurement Information System; PSS, Perceived Stress Scale; TNF, Tumor Necrosis Factor.

* *p* < 0.05.

** *p* < 0.01.

### Serial-Mediation analyses

**Figure 2:**
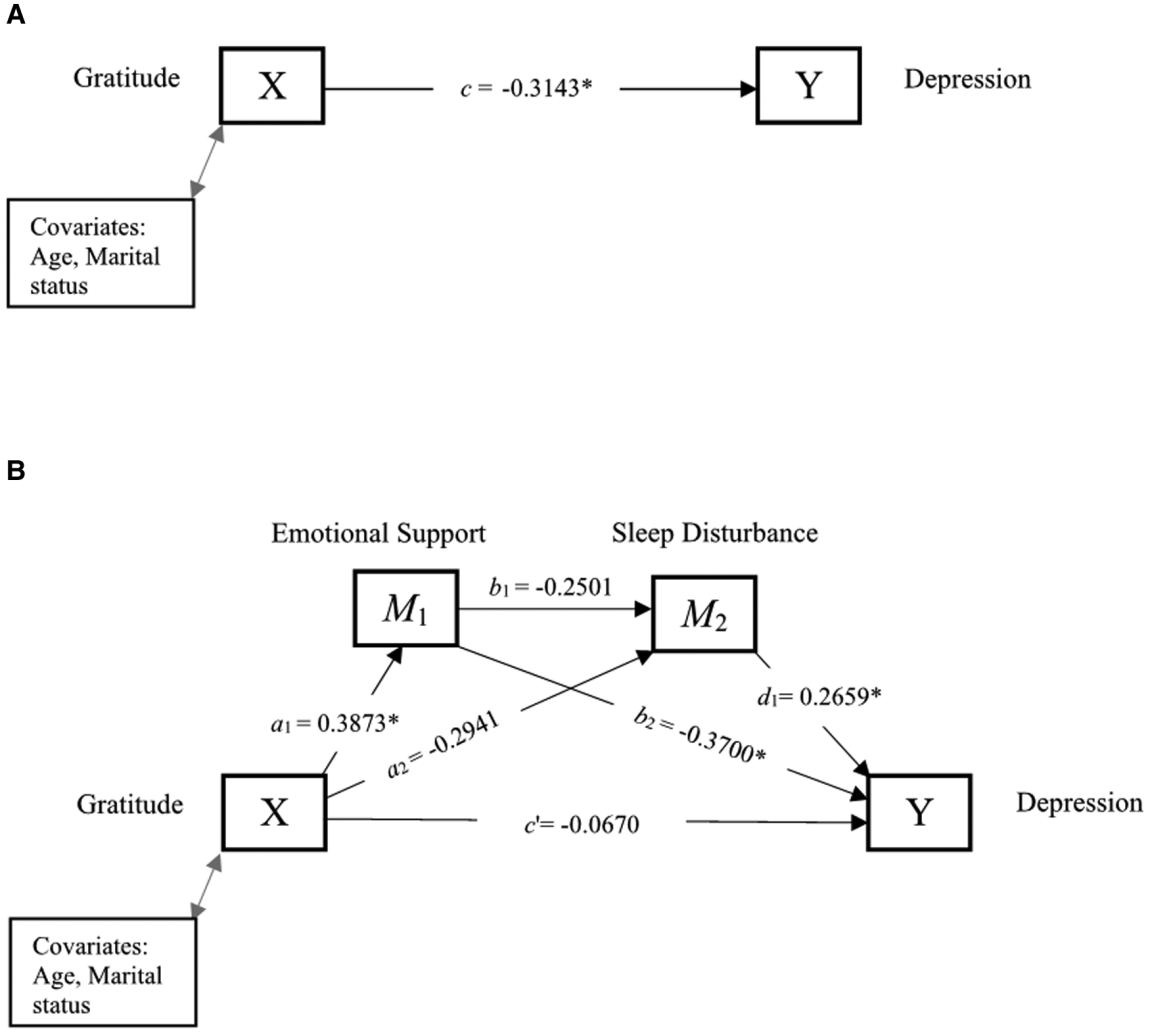
Serial mediation of social support and sleep disturbance. (**A**) Path diagram showing the total effect of gratitude on depression. (**B**) Serial mediation model of the direct and indirect effects of gratitude on depression through social support and sleep disturbance. *p < 0.05 is statistically significant.

Figure 2 and Table 3 show the total effect, and direct and indirect effects of gratitude and depression through emotional support and sleep disturbance. The total effect, *c*, of gratitude (X) on depression (Y) after controlling for covariates was −0.3143, indicating higher levels of gratitude significantly predict lower depression. The direct effect *c*′ was not statistically significant, indicating that gratitude was unrelated to depression, independent of the effect of emotional support and sleep disturbance. The indirect effects, *a*_1_*b*_2_, and *a*_1_*b*_1_*d*_1_, were not significant. However, the indirect effect (*a*_2_*d*_1_) of gratitude on depression through sleep was significant (95% CI: −0.2094 to −0.0055), suggesting that those who experienced greater gratitude experienced less sleep disturbance (*a*_2_*_ _*= − 0.2941), which in turn was associated with lower depression (*d*_1_*_ _*= 0.2659).

**Table 3:** Direct and indirect effects between gratitude and depression through emotional support and sleep disturbance.

Path	Estimate	95% CI	
		Lower	Upper
Total effect (*c*)	−0.3143*	−0.5170	−0.1117
Direct effect (*c*′)	−0.0670	−0.2318	0.0978
*a*_1 (Gratitude → Emotional Support)_	0.3873*	0.1130	0.6617
*a*_2 (Gratitude → Sleep Disturbance)_	−0.2941	−0.5915	0.0032
*b*_1 (Emotional Support → Sleep Disturbance)_	−0.2501	−0.5216	0.0213
*b*_2 (Emotional Support → Depression)_	−0.3700*	−0.5198	−0.2202
*d*_1 (Sleep disturbance → Depression)_	0.2659*	0.1212	0.4106
**Indirect effects**			
*a*_1_ *b*_2_	−0.1433	−0.3246	0.0059
*a*_2_ *d*_1_	−0.0782	−0.2094	−0.0055
*a*_1_ *b*_1_ *d*_1_	−0.0258	−0.0825	0.0123
Total indirect effect	−0.2473	−0.4671	−0.0649

* *p* < 0.05 is statistically significant.

**Figure 3:**
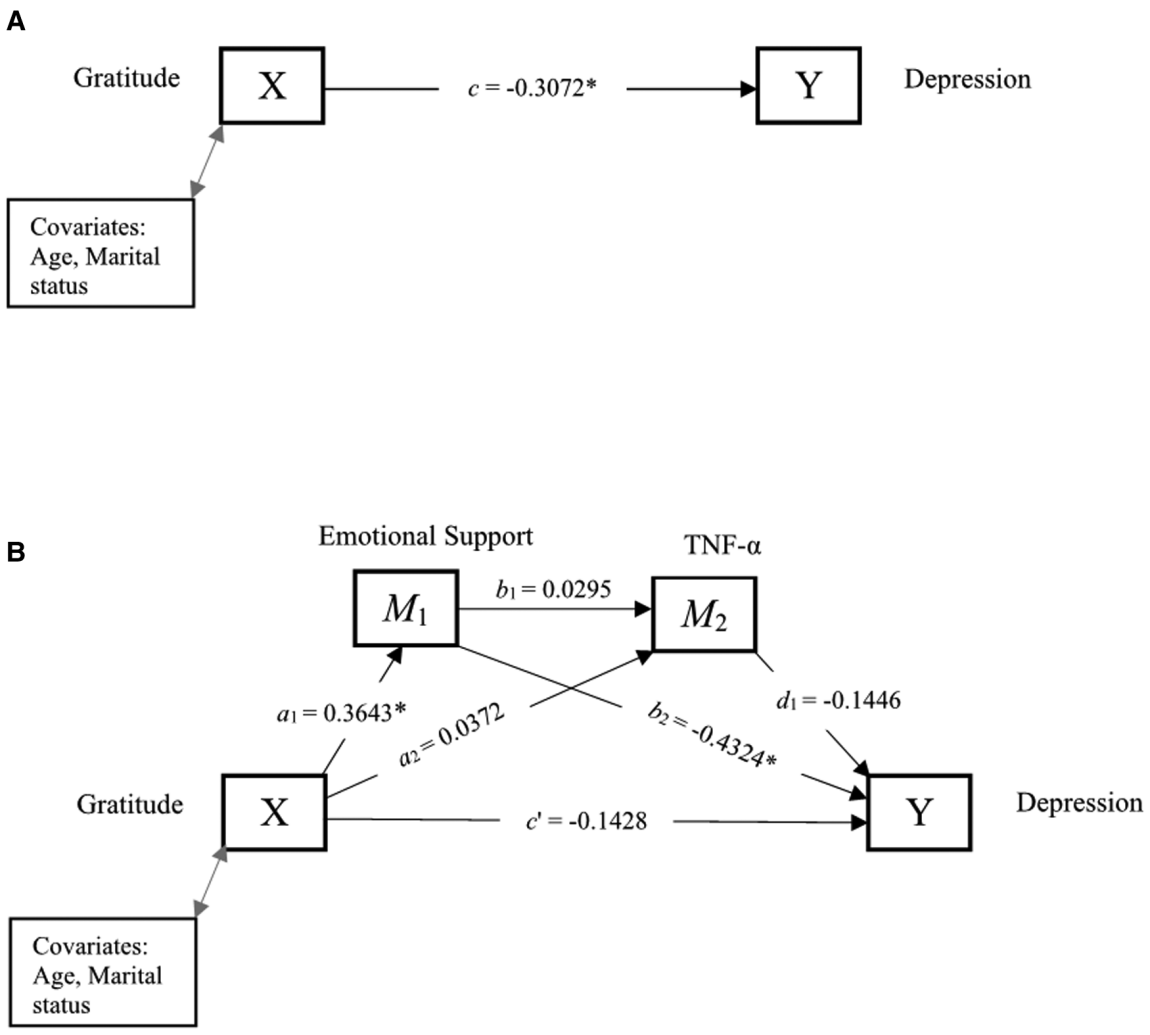
Serial mediation of social support and TNF-α. (**A**) Path diagram showing the total effect of gratitude on depression. (**B**) Serial mediation model of the direct and indirect effects of gratitude on depression through emotional support and TNF-α. *p < 0.05 is statistically significant.

Figure 3 and Table 4 show the total effect, and direct and indirect effects of gratitude and depression through emotional support and TNF-α. The total effect, *c*, of gratitude (X) on depression (Y) after controlling for covariates was −0.3072, indicating that higher levels of gratitude were significantly associated with lower depression. The direct effect *c*′ was not statistically significant, suggesting that gratitude was unrelated to depression after including emotional support and TNF-α in the model. Further, the indirect effects (*a*_1_*b*_2_, *a*_2_*d*_1_, *a*_1_*b*_1_*d*_1_) were all non-significant.

**Table 4:** Direct and indirect effects between gratitude and depression through social support and TNF-α.

Path	Estimate	95% CI	
		Lower	Upper
Total effect (*c*)	−0.3072*	−0.5225	−0.0920
Direct effect (*c*′)	−0.1428	−0.3329	0.0473
*a*_1__(Gratitude → Emotional Support)_	0.3643*	0.0760	0.6526
*a*_2 (Gratitude → TNF-α)_	0.0372	−0.0518	0.1262
*b*_1 (Emotional Support → TNF-α)_	0.0295	−0.0523	0.1112
*b*_2 (Emotional Support → Depression)_	−0.4324*	−0.6068	−0.2580
*d*_1 (TNF-α → Depression)_	−0.1446	−0.7473	0.4580
**Indirect effects**			
*a*_1_ *b*_2_	−0.1575	−0.3596	0.0316
*a*_2_ *d*_1_	−0.0054	−0.0420	0.0268
*a*_1_ *b*_1_ *d*_1_	−0.0016	−0.0175	0.0077
Total indirect effect	−0.1645	−0.3679	0.0329

* *p* < 0.05 is statistically significant.

**Figure 4:**
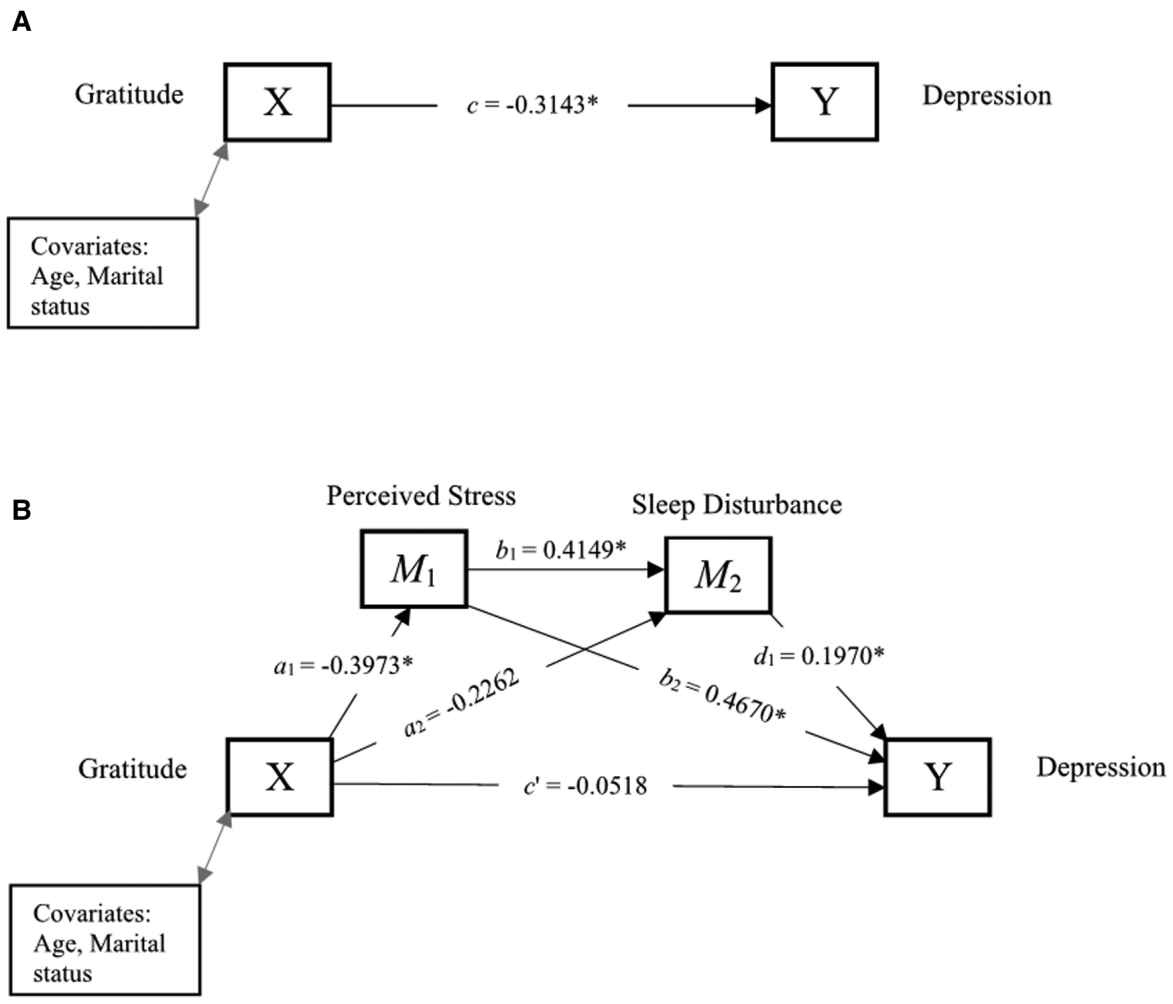
Serial mediation of perceived stress and sleep disturbance. (**A**) Path diagram showing the total effect of gratitude on depression. (**B**) Serial mediation model of the direct and indirect effects of gratitude on depression through perceived stress and sleep disturbance. *p < 0.05 is statistically significant.

Figure 4 and Table 5 show the total effect, and direct and indirect effects of gratitude and depression through perceived stress and sleep disturbance. The total effect, *c*, of gratitude (X) on depression (Y) after controlling for covariates was −0.3143, indicating higher levels of gratitude predict lower depression. The direct effect *c*′, was not statistically significant, suggesting that gratitude was unrelated to depression, independent of the effect of perceived stress and sleep disturbance. Further, the indirect effect (*a*_2_*d*_1_) of gratitude on depression through sleep disturbance was not significant. However, the indirect effect of gratitude on perceived stress and sleep disturbance in serial, with perceived stress modeling as affecting sleep disturbance, which in turn influences depression (*a*_1_*b*_1_*d*_1_) was significant (95% CI: −0.0970 to −0.0020). Those who experienced higher gratitude experienced lower perceived stress (*a*_1_ = − 0.3973), which was associated with lower sleep disturbance (*b*_2_ = 0.4149), and which in turn was associated with lower depression (*d*_1 _= 0.1970). The indirect effect (*a*_1_*b*_2_) of gratitude on depression through perceived stress was also significant (95% CI: −0.3735 to −0.0375), indicating that those who experienced greater gratitude had lower perceived stress (*a*_1_ = − 0.3973), which in turn was associated with lower depression (*b*_2_ = 0.4670).

**Table 5:** Direct and indirect effects between gratitude and depression through perceived stress and sleep disturbance.

Path	Estimate	95% CI	
		Lower	Upper
Total effect (*c*)	−0.3143*	−0.5170	−0.1117
Direct effect (*c*′)	−0.0518	−0.2070	0.1035
*a*_1 (Gratitude → Perceived Stress)_	−0.3973*	−0.6515	−0.1430
*a*_2 (Gratitude → Sleep Disturbance)_	−0.2262	−0.5147	0.0623
*b*_1 (Perceived Stress → Sleep Disturbance)_	0.4149*	0.1347	0.6951
*b*_2 (Perceived Stress → Depression)_	0.4670*	0.3081	0.6259
*d*_1 (Sleep Disturbance→ Depression)_	0.1970*	0.0547	0.3393
**Indirect effects**			
*a*_1_ *b*_2_	−0.1855	−0.3735	−0.0375
*a*_2_ *d*_1_	−0.0446	−0.1316	0.0081
*a*_1_ *b*_1_ *d*_1_	−0.0325	−0.0970	−0.0020
Total indirect effect	−0.2626	−0.5008	−0.0858

* *p* < 0.05 is statistically significant.

**Figure 5:**
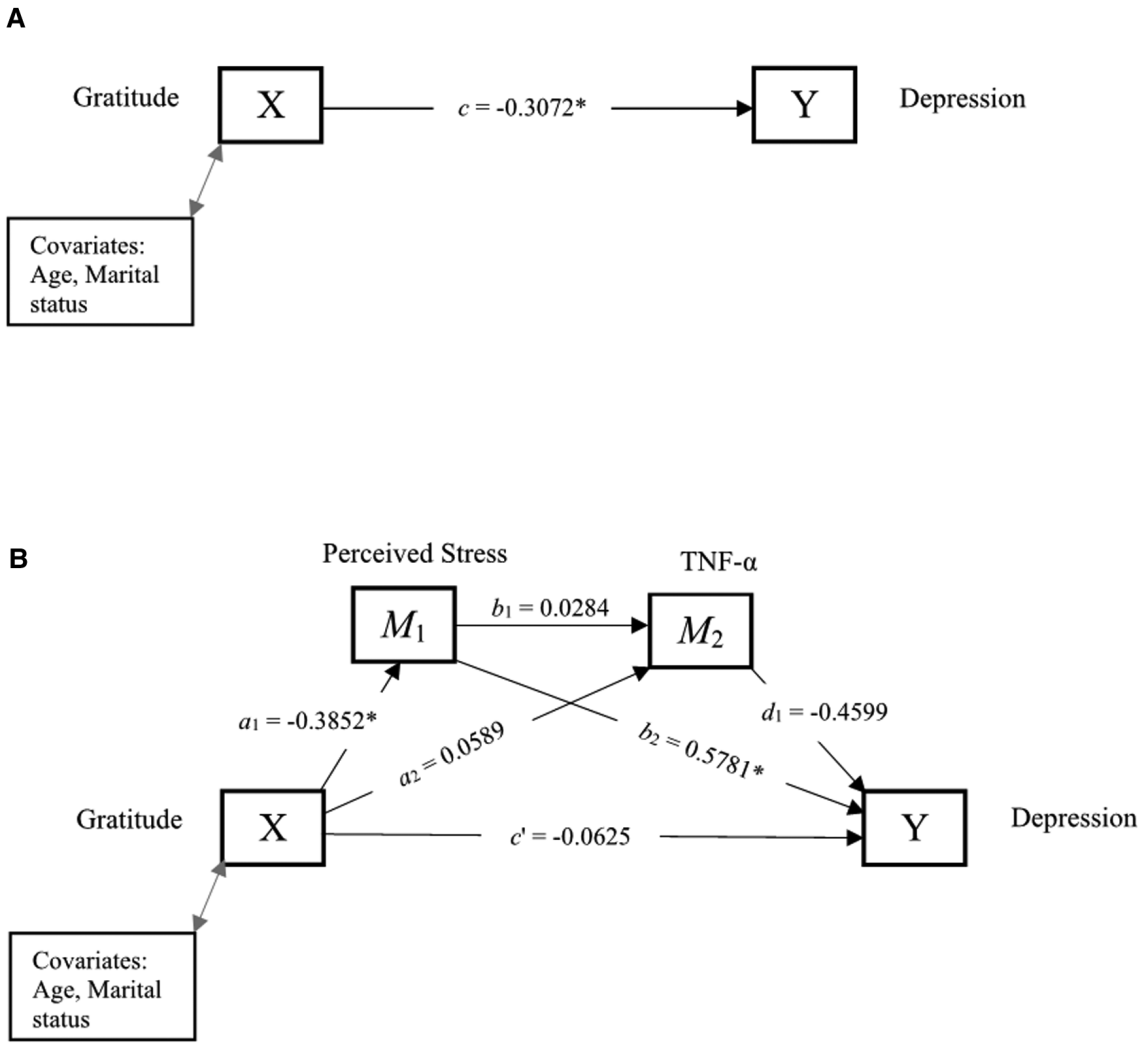
Serial mediation of perceived stress and TNF-α. (**A**) Path diagram showing the total effect of gratitude on depression. (**B**) Serial mediation model of the direct and indirect effects of gratitude on depression through perceived stress and TNF-α. *p < 0.05 is statistically significant.

Figure 5 and Table 6 show the total effect, and direct and indirect effects of gratitude and depression through perceived stress and TNF-α. The total effect, *c*, of gratitude (X) on depression (Y) after controlling for covariates was −0.3072, indicating that higher levels of gratitude were significantly associated with lower depression. The direct effect *c*′ was not statistically significant, suggesting that gratitude was unrelated to depression after including perceived stress and TNF-α into the model. Further, the indirect effects (*a*_2_*d*_1_, and *a*_1_*b*_1_*d*_1_) were not significant. However, the indirect effect (*a*_1_*b*_2_) of gratitude on depression through perceived stress was significant (95% CI: −0.4931 to −0.0293). Experiences of gratitude were related to lower levels of perceived stress (*a*_1_*_ _*= −0.3852), which in turn was associated with lower levels of depression (*b*_2_*_ _*= 0.5781).

**Table 6:** Direct and indirect effects between gratitude and depression through perceived stress and TNF-α.

Path	Estimate	95% CI	
		Lower	Upper
Total effect (*c*)	−0.3072*	−0.5225	−0.0920
Direct effect (*c*′)	−0.0625	−0.2320	0.1070
*a*_1 (Gratitude → Perceived Stress)_	−0.3852*	−0.6483	−0.1221
*a*_2 (Gratitude → TNF-α)_	0.0589	−0.0319	0.1497
*b*_1 (Perceived Stress → TNF-α)_	0.0284	−0.0613	0.1181
*b*_2 (Perceived Stress → Depression)_	0.5781*	0.4127	0.7435
*d*_1 (TNF-α → Depression)_	−0.4599	−0.9814	0.0617
**Indirect effects**			
*a*_1_ *b*_2_	−0.2227	−0.4931	−0.0293
*a*_2_ *d*_1_	−0.0271	−0.0913	0.0174
*a*_1_ *b*_1_ *d*_1_	0.0050	−0.0090	0.0435
Total indirect effect	−0.2447	−0.5186	−0.0398

* *p* < 0.05 is statistically significant.

## Discussion

To our knowledge, this is the first study to examine the mechanisms underlying the association between gratitude and depression in older adults with cLBP. There is a growing interest in the value of gratitude in improving well-being in painful chronic health conditions, with evidence suggesting that gratitude plays an important role in reducing depressive symptoms (54, 55). Consistent with our hypotheses, gratitude was correlated with lower levels of perceived stress, depression, and sleep disturbance, and greater levels of perceived social support. Conversely, gratitude was not significantly associated with TNF-α. Importantly, we found that perceived stress and sleep disturbance serially mediate the association between gratitude and depression, even after controlling for age and marital status. Overall, results are in accordance with prior work in chronic illness populations linking higher levels of gratitude to decreased stress and depression, as well as greater perceived social support and sleep quality (35, 56, 57). These findings align with current theoretical models of positive emotion (58) signifying that gratitude may promote adaptive health outcomes through the broadening of behavioral and cognitive activities that bolster physical, intellectual, and psychosocial resources.

Supporting the tenets of the PPWB model (22, 23), mediation analyses revealed that perceived stress and sleep disturbance might serve as mechanisms underpinning the link between gratitude and psychological well-being. In the context of these findings, people with higher levels of gratitude may have a greater propensity to engage in protective health behaviors (e.g., positive cognitions, exercise, healthy diet) that promote resilience. For instance, grateful people may be more likely to treat themselves with compassion and support and have higher self-esteem when setbacks occur, thereby reducing symptoms of depression (59, 60). Gratitude may also cultivate positive emotions that mitigate the impact of stress and increase positive outlook on life despite the presence of chronic illness (19, 35). As such, those who perceive lower levels of stress in their lives may be less susceptible to negative pre-sleep cognitions (i.e., negative and worrying thoughts) that facilitate sleep disturbance (56, 61). Indeed, it has been suggested that stress increases arousal of cognitive, somatic, and cortical activation during the pre-sleep period, an effect which negatively influences overall sleep quality and serves as a risk factor for the development of depression (62).

Grateful people are also more likely to have greater perceptions of social support (63, 64) and behave pro-socially to express their gratitude which helps to strengthen social bonds and build social resources (58). Evidence suggests that high levels of social support are associated with less sleep disturbance and lower inflammation in individuals with chronic low back pain (65), as well as in older adults (66). In support of these studies, correlational analyses revealed that individuals who reported greater social support also reported significantly less sleep disturbance. Surprisingly, our findings did not support the mediating role of social support in the aforementioned relationships. However, it is important to note that the quantity of social support as well as the type of supportive resource may have differential influences on health-related outcomes. Future studies should consider examining other areas of social support beyond emotional resources such as informational (e.g., advice, feedback) and instrumental (e.g., materials, actions) methods, and/or use social support measures that are more relevant to pain than general support measures in daily-life situations.

Despite attempts at identifying biological pathways by which gratitude may lessen negative mood, TNF-α did not account for this relationship and gratitude was not significantly correlated with TNF-α. While limited research has examined the effects of gratitude on health using inflammatory biomarkers, relationships between positive psychological factors and inflammation have been less consistent as compared to associations with health behaviors (e.g., smoking, alcohol consumption, exercise, diet, medication adherence) (67, 68). Nevertheless, there is preliminary evidence suggesting a possible association between positive psychological processes (e.g., positive affect, gratitude) and lower levels of inflammatory markers (e.g., TNF-α, interleukin-6, C-reactive protein) (69, 70) in individuals with cardiovascular disease. Additional research considering other physiological factors (e.g., interleukin-6) not tested here is warranted in older adults with chronic pain.

### Clinical implications

Findings from the current study have potential clinical relevance. Specifically, therapeutic approaches designed to promote gratitude may be advantageous to improve well-being in older adults with cLBP. Indeed, evidence suggests that gratitude interventions improve subjective well-being, overall health, depression, and perceived stress levels (71–75). Although not extensively studied, gratitude interventions also show promise in improving physical health outcomes, including sleep quality and levels of inflammation (76). For example, a recent randomized-controlled trial found that women with emotional distress and sleep disturbance who kept a gratitude journal for two weeks had greater increases in hedonic well-being, optimism, and sleep quality, relative to those who wrote about everyday events (77). Thus, interventions aimed at augmenting gratitude may have positive downstream effects on stress and sleep efficiency and have particular therapeutic efficacy for older adults with comorbid pain and depression.

### Limitations and future directions

These findings should be considered in light of their limitations. First, the sample size was small and included older adults with cLBP who were largely White/Caucasian (70%) and unemployed (85%). The generalizability of the findings would be improved through replication in other chronic pain samples from diverse populations. Second, this study used a cross-sectional design; therefore, causal relationships cannot be inferred. Future prospective studies would be warranted to explore the long-term effects of gratitude on depression and the potential health and biological factors mediating this relationship. Third, it is unclear whether results will replicate with objective sleep indices as we only included a self-report measure of sleep disturbance. Fourth, while our testing procedures were scheduled within a restricted time window, careful consideration of the timing of questionnaires and sampling of TNF-α is warranted in future work given evidence of diurnal fluctuations in inflammatory cytokines and affective states (78–81). Despite these limitations, the present study makes several important contributions. To our knowledge, this is the first study using the PPWB model to examine the mechanisms underlying the association between gratitude and depression, and the first to examine gratitude in community-dwelling older adults with cLBP. The study also used valid and reliable measures to assess study variables. Despite the small sample size, the present study provides a foundation for future research to explore gratitude as a potential resilience factor and examine the therapeutic benefit of gratitude activities on health and well-being among older adults with chronic pain.

## Conclusion

In sum, findings suggest that perceived stress and sleep disturbance may be important mechanisms contributing to the link between gratitude and depression in older adults with cLBP. This causal pathway should be confirmed by longitudinal studies with a larger sample. The consideration and integration of gratitude interventions into new or existing therapeutic modalities may be a step toward optimizing mental and physical health in older adults with chronic pain.

## Data Availability

The raw data supporting the conclusions of this article will be made available by the authors, without undue reservation.
